# Prescribing Patterns and Adverse Reactions of Antipsychotics in a North Indian Psychiatry Outpatient Department

**DOI:** 10.7759/cureus.96281

**Published:** 2025-11-07

**Authors:** Shivani Sodhi, Himanshu Singh, Amit Kumar Sharma, Shaily Mina, Sparsh Gupta, Nidhi P Vadanere

**Affiliations:** 1 Department of Pharmacology, Lady Hardinge Medical College and Associated Smt. Sucheta Kriplani (SSK) and Kalawati Saran Children (KSC) Hospitals, New Delhi, IND; 2 Department of Pharmacology, Vardhman Mahavir Medical College and Safdarjung Hospital, New Delhi, IND; 3 Department of Psychiatry, Vardhman Mahavir Medical College and Safdarjung Hospital, New Delhi, IND; 4 Department of Community Medicine, Lady Hardinge Medical College and Associated Smt. Sucheta Kriplani (SSK) and Kalawati Saran Children (KSC) Hospitals, New Delhi, IND

**Keywords:** antipsychotics, drug utilization, pharmacovigilance, polypharmacy, prescription patterns, schizophrenia

## Abstract

Background: Pharmacovigilance research in India remains at an infantile phase, especially in the pattern of prescription of antipsychotics at the tertiary care level. Given the clinical value of antipsychotic drugs, research on their utilization, trends of prescription, and frequent adverse events associated with them will help to reduce adverse drug events. The proposed study is thus intended to discuss the prescription pattern and adverse drug reactions (ADRs) of antipsychotic drugs in the Psychiatric Outpatient unit in Vardhman Mahavir Medical College and Safdarjung Hospital, New Delhi, one of the tertiary care hospitals in North India.

Methods: In this cross-sectional investigation, systematic random sampling with a fixed sampling interval was used, wherein every third patient attending the Psychiatry Outpatient Department during the study period was selected until the desired sample size of 150 participants (aged 18-65 years) was achieved. The following statistics were collected: medication history, antipsychotic prescriptions, adverse drug events, diagnosis (according to Diagnostic and Statistical Manual of Mental Disorders, Fifth Edition (DSM-5) criteria and definition), and sociodemographics. The information gathered was utilized to ascertain treatment compliance, antipsychotic combinations, and prescription trends as reported by the World Health Organization (WHO) in the Drug Prescribing Indicators.

Results: Sixty-nine (46.3%) patients in this research received antipsychotic medications under generic names, while 13 (8.7%) received several antipsychotic medications. With a mean ± SD of 2.69 ± 0.84 medications per encounter and 1.13 ± 0.32 antipsychotics per patient, the prescribing trend indicated limited polypharmacy. The most widely used antipsychotics were risperidone in 63 (42%) patients, primarily prescribed for schizophrenia and psychosis not otherwise specified (NOS), followed by olanzapine in 41 (27.3%), commonly used in bipolar affective disorder and schizophrenia. There was a total of 150 study participants, among whom 42 adverse reactions were recorded. The most common ADR was akathisia in nine (21.4%), followed by tremors in six (14.3%) and drowsiness in six (14.3%). According to the WHO/Uppsala Monitoring Centre (UMC) causality assessment approach, over 90.5% of adverse drug responses could be identified as potential ADRs, and roughly 9.5% were likely ADRs.

Conclusion: It is necessary to justify the antipsychotic drug prescribing pattern. By evaluating prescription habits, one may determine the incidence of pharmacovigilance and polytherapy, which helps to minimize and avoid adverse medication responses.

## Introduction

Antipsychotic medications are essential in the management of schizophrenia, bipolar affective disorder, and other psychotic illnesses [1]. These disorders are characterized by delusions, hallucinations, disorganized thought, and behavioral disturbances that significantly affect quality of life and healthcare expenditure [2]. Over the past two decades, psychopharmacological management has become the mainstay of therapy, with atypical (second-generation) antipsychotics such as risperidone, olanzapine, aripiprazole, quetiapine, and clozapine largely replacing typical agents like haloperidol due to their better safety profile [3].

Monitoring prescribing patterns is vital to ensure rational use, prevent polypharmacy, and minimize adverse reactions [4]. Prescription-based studies serve as important tools to evaluate physicians’ prescribing behavior and identify deviations from standard guidelines [5]. Drug utilization research helps understand how medications are prescribed and used, thereby improving patient safety and optimizing treatment outcomes [6,7].

The present study aimed to evaluate the prescribing patterns and adverse reactions associated with antipsychotic medications in the psychiatry outpatient department of a tertiary care hospital in North India. By identifying commonly prescribed drugs and their indications, this study seeks to promote rational antipsychotic use and strengthen pharmacovigilance practices in psychiatric care.

The objectives of the present study were (1) to evaluate the prescription patterns of antipsychotic medications among psychiatric outpatients in a tertiary care hospital in North India, (2) to identify and assess adverse drug reactions (ADRs) associated with the use of antipsychotic medications, and (3) to analyze the World Health Organization (WHO) core prescribing indicators and assess the rationality of prescriptions in terms of polypharmacy, generic prescribing, and adherence to the essential medicines list.

## Materials and methods

Study design and setting

This descriptive cross-sectional study was conducted in the Psychiatry Outpatient Department (OPD) of Vardhman Mahavir Medical College and Safdarjung Hospital, New Delhi, in collaboration with the Department of Pharmacology. The study was carried out between June 2022 and December 2023, adhering to institutional and national ethical standards and the principles of the Declaration of Helsinki.

The Population (P) is adult patients aged 18-65 years attending the Psychiatry OPD and prescribed at least one antipsychotic medication. The Intervention (I) is prescription and monitoring of antipsychotic medications, including both typical and atypical agents. The Comparison (C) is patterns of prescribing across different patient groups and combinations of antipsychotic use. The Outcome (O) is the evaluation of prescribing patterns, adherence to WHO prescribing indicators, and identification of associated adverse reactions.

The study thus aimed to assess trends in antipsychotic prescribing and their related adverse effects to promote rational drug use and enhance pharmacovigilance practices.

Study population

The study population comprised adult patients attending the Psychiatry OPD who were prescribed at least one antipsychotic drug during the study period. Each eligible patient was approached during their visit, the purpose of the study was explained, and written informed consent was obtained before inclusion. Patients were then selected using systematic random sampling until the desired sample size was achieved.

Eligibility criteria

Participants were eligible if they were between 18 and 65 years of age, had a psychiatric diagnosis confirmed according to DSM-5 criteria by the treating psychiatrist, and were prescribed at least one antipsychotic medication, whether newly initiated or continued. Patients who were unable to provide informed consent or who had uncontrolled or unstable medical conditions that could interfere with participation were excluded from the study. Prescriptions with incomplete data were also excluded.

Sample size and sampling technique

The final sample size was 150 encounters. The number was determined using the methodology recommended in the World Health Organization (WHO)'s manual on how to investigate drug use in health facilities, which recommends a minimum of 100-200 encounters to obtain stable estimates of prescribing indicators. This sample size provided a balance between statistical precision and operational feasibility in a high-volume outpatient setting. Patients were recruited using systematic random sampling, ensuring equal probability of selection from the OPD attendance list on data collection days [8].

Study parameters

Data were collected using a pretested case record form, which captured sociodemographic variables including age, sex, educational status, occupation, and family type. Clinical information recorded included the primary psychiatric diagnosis as per DSM-5 criteria. Detailed prescription data were noted for each encounter, including drug name (generic or brand), dose, frequency, route, formulation, and whether the drug appeared in the National List of Essential Medicines (NLEM 2022). Prescription patterns were analyzed according to the WHO prescribing indicators, including the average number of drugs per encounter, percentage of drugs prescribed by generic name, percentage of encounters with injections, percentage of drugs from the essential medicines list, average number of antipsychotics per encounter, and percentage of encounters with more than one antipsychotic prescribed. Concomitant psychotropics such as benzodiazepines, anticholinergics, mood stabilizers, and antidepressants were also documented.

ADRs observed or reported during the encounter were recorded using a standard Adverse Drug Event Reporting Form. Each ADR was evaluated using the WHO-UMC causality assessment system and categorized as possible, probable, or certain [9]. The spectrum of ADRs included extrapyramidal symptoms such as akathisia and tremors, sedation, weight gain, hypersalivation, and other clinically significant events.

Data collection and management

All data were collected contemporaneously during patient visits to minimize recall bias and ensure completeness. Completed forms were entered into Microsoft Excel (Microsoft Corp., USA), with de-identified patient codes to maintain confidentiality. Data accuracy was verified by cross-checking a random subset of entries against the original records. Access to the electronic dataset was restricted to the study investigators.

Statistical analysis

The data were analyzed using IBM SPSS Statistics for Windows, version 21.0 (released 2012, IBM Corp., Armonk, NY). Continuous variables such as the number of drugs per encounter were expressed as mean ± standard deviation, while categorical variables such as sex distribution, diagnosis, and ADR frequencies were summarized as n (%). Prescribing indicators were calculated according to the WHO methodology, and 95% confidence intervals were estimated for key proportions where applicable. Descriptive statistics formed the basis of the analysis, with exploratory comparisons made using Chi-square tests for categorical variables and t-tests or ANOVA for continuous variables when relevant. A two-sided significance level of 0.05 was applied for inferential testing.

Ethics approval

Before initiation, the study protocol was reviewed and approved by the Institutional Ethics Committee of Vardhman Mahavir Medical College and Safdarjung Hospital (no. IEC/VMMC/SJH/Thesis/06/2022/CC-241). Written informed consent was obtained from all participants before data collection commenced.

## Results

The mean age was 36.4 ± 11.5 years, with 52 (34.7%) belonging to the group of 18-30 years. The female population comprised 85 (56.7%), compared to 65 (43.3%) males. The majority of the patients were residing in a joint family (n = 97, 64.7%) and were unemployed (n = 81, 54.0%). Approximately 49 (32.7%) were not educated, 33 (22.0%) had reached the middle level of education, and approximately 68 (45.3%) had completed high school or higher. Thirteen (8.7%) participants were prescribed more than one antipsychotic. Most of these prescriptions included olanzapine with aripiprazole in nine (6%) cases, followed by risperidone with aripiprazole in four (2.7%). According to the WHO/UMC causality evaluation approach, 38 (90.5%) of the 42 ADRs were determined to be conceivable in nature, and four (9.5%) were likely. Table 1 summarizes the sociodemographic characteristics of the 150 participants. The majority of patients were females (n = 85, 56.7%) and belonged to joint families (n = 97, 64.7%). Most participants were unemployed (n = 81, 54.0%), and a considerable proportion were illiterate (n = 49, 32.7%), while 68 (45.3%) had completed high school or higher education. The largest age group represented was 18-30 years, comprising 52 (34.7%) of the sample.

**Table 1:** Sociodemographic characteristics of the study population (N = 150)

Sno	Sociodemographic details	Male (n, %)	Female (n, %)	Total
I	Age groups (in completed years)	-	-	-
1	18-30 years	21 (40.4)	31 (59.62)	52
2	31-40 years	25 (52.1)	23 (47.9)	48
3	41-50 years	10 (34.5)	19 (65.5)	29
4	51-60 years	04 (30.8)	09 (69.2)	13
5	>60 years	05 (62.5)	03 (37.5)	08
III	Educational status	-	-	-
1	Illiterate	15 (30.6)	34 (69.4)	49
2	Middle class	14 (42.4)	19 (57.6)	33
3	High school	21 (48.8)	22 (51.2)	43
4	Higher secondary school	10 (71.4)	04 (28.6)	14
5	Graduate and above	05 (45.5)	06 (54.5)	11
IV	Occupation	-	-	-
1	Unemployed	18 (22.2)	63 (77.8)	81
2	Employed	47 (68.1)	22 (31.9)	69
V	Type of family	-	-	-
1	Nuclear	23 (43.4)	30 (56.6)	53
2	Joint	42 (43.3)	55 (56.7)	97

**Figure 1:**
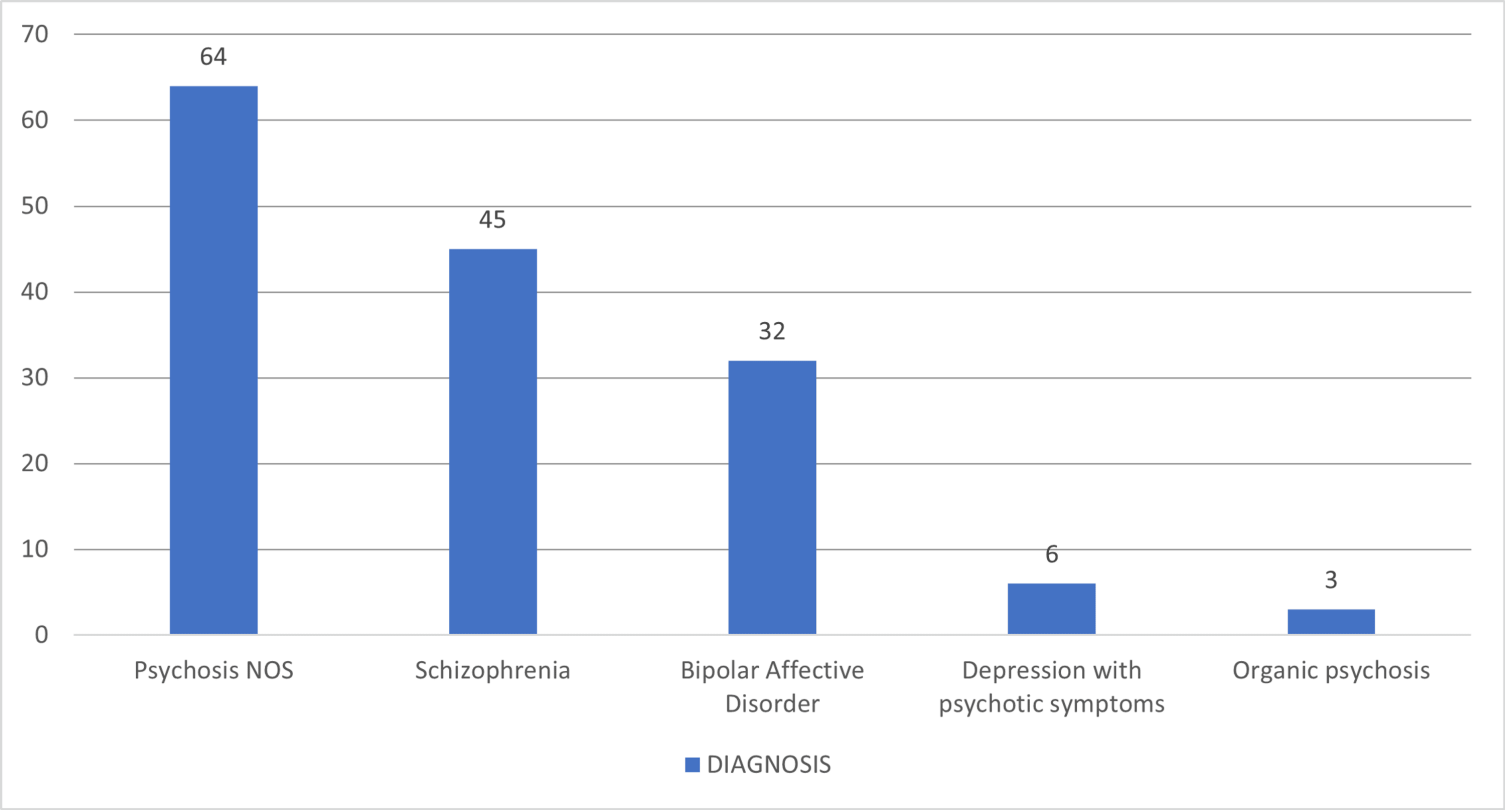
Distribution of study participants according to the clinical profile (N = 150)

Figure 1 depicts the diagnostic profile of the study population. Psychosis NOS was the most common diagnosis, present in 64 (42.7%) patients, followed by schizophrenia in 45 (30%) and bipolar affective disorder in 32 (21.3%). Less frequent diagnoses included depression with psychotic symptoms (n = 6, 4%) and organic psychosis (n = 3, 2%). This distribution highlights the predominance of psychotic spectrum disorders in the outpatient cohort.

**Figure 2:**
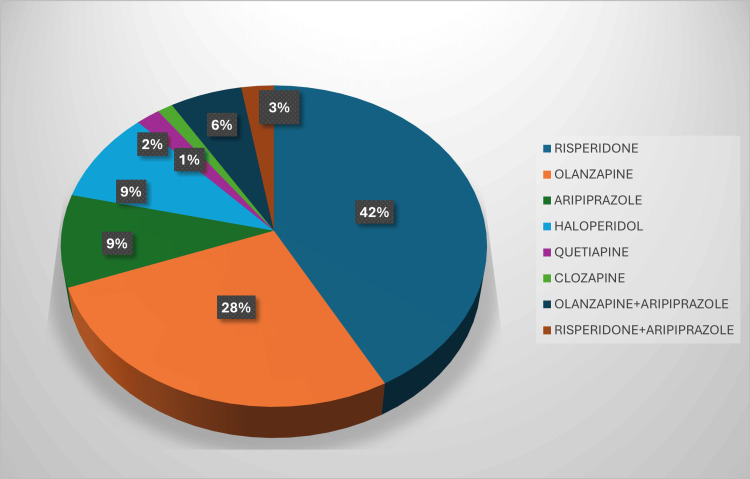
Prescribing pattern of antipsychotic drugs in the study population (N = 150)

Figure 2 illustrates the prescribing trends for antipsychotic medications. Risperidone was the most frequently prescribed antipsychotic, accounting for 63 (42%) prescriptions, followed by olanzapine with 41 (27.3%). Aripiprazole and haloperidol were each used in 14 (9.3%) prescriptions, while quetiapine and clozapine were less commonly prescribed at three (2%) and two (1.3%), respectively. This distribution indicates a strong preference for atypical antipsychotics in routine practice.

Table 2 presents the results of the WHO drug prescribing indicators. The mean number of drugs per encounter was 2.69 ± 0.84, which is slightly higher than the WHO optimal range and may indicate a tendency toward polypharmacy. A total of 69 (46.3%) prescriptions were written using generic names, which falls short of the WHO recommendation of 100%, highlighting a reliance on brand prescribing. Poly-antipsychotic use was identified in 13 (8.7%) encounters, while injectable formulations were prescribed in six (4%), well within the WHO-recommended range. Furthermore, 109 (72.5%) of the prescribed drugs were from the NLEM, suggesting moderate adherence to essential medicine use but with room for improvement.

**Table 2:** Antipsychotic prescribing pattern as per the World Health Organization (WHO) Drug Prescribing Indicators

Indicator	Result (Mean ± SD / %)	Interpretation
Average number of drugs per encounter	2.69 ± 0.84	Slightly higher than the WHO optimal range (1.6–1.8), suggesting a mild tendency toward polypharmacy. This may partly be attributed to the concomitant use of centrally acting agents such as benzodiazepines and opioid analgesics like tramadol, which are occasionally prescribed for comorbid symptoms such as anxiety, insomnia, or chronic pain, and can produce similar sedative and central nervous system effects.
Average number of antipsychotics per encounter	1.13 ± 0.32	Indicates that most encounters involved a single antipsychotic, although a small proportion reflected polyantipsychotic use
Percentage of drugs prescribed by generic name	46.3%	Well below the WHO recommendation of 100%, suggesting a reliance on brand-name prescribing
Percentage of encounters with >1 antipsychotic prescribed	8.7%	Indicates poly-antipsychotic use in nearly one in 12 encounters, which may not align with rational prescribing practices
Percentage of encounters with an antibiotic prescribed	0%	Acceptable, as antibiotics are not typically indicated in psychiatric encounters
Percentage of encounters with an injection prescribed	4.0%	Within the WHO-recommended range (<10–15%), indicating limited use of parenteral formulations
Percentage of drugs from the essential drug list/formulary	72.5%	Below the WHO target of 100%, highlighting the need to improve adherence to the National List of Essential Medicines (NLEM 2022)

**Figure 3:**
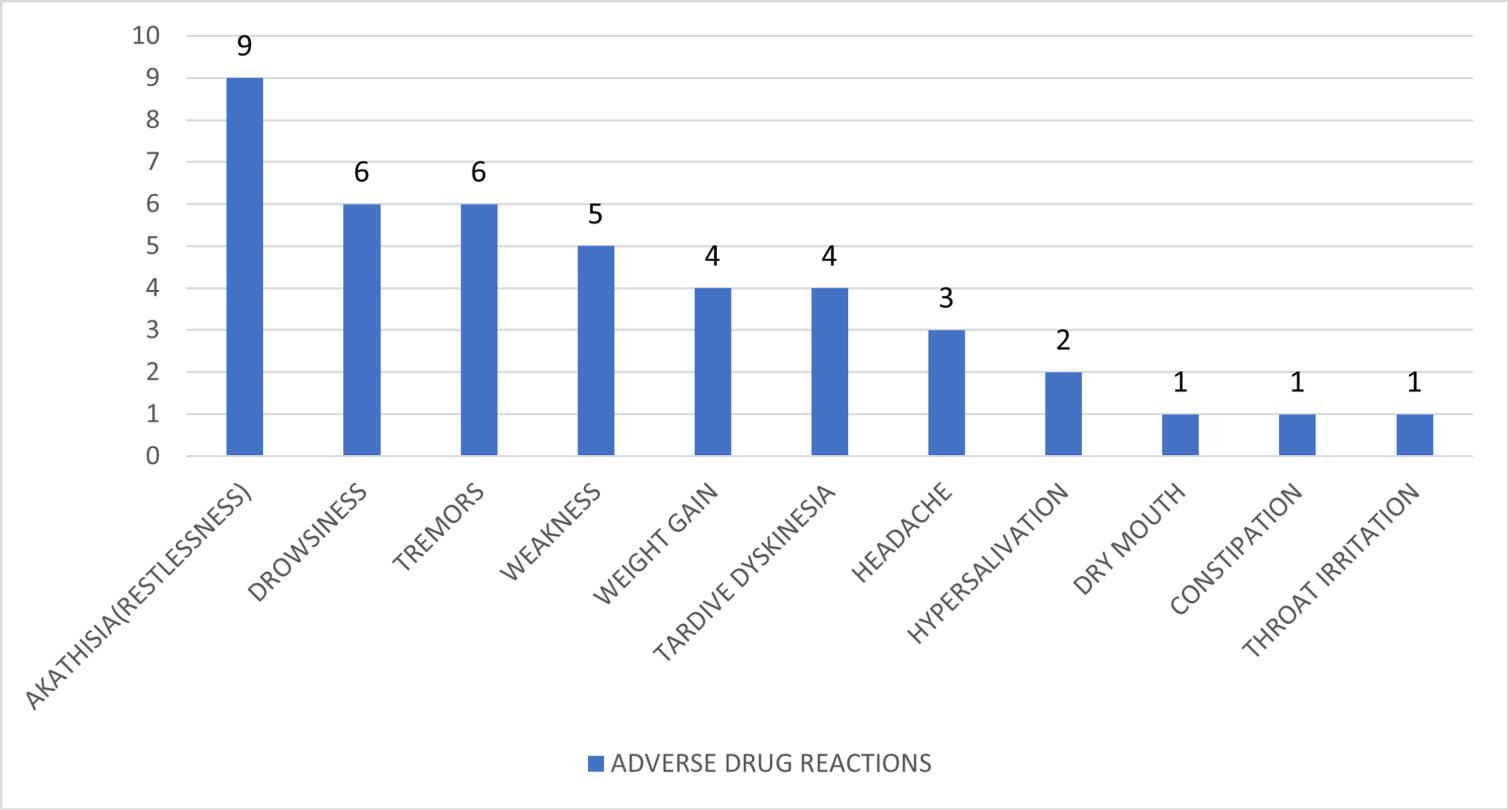
Adverse drug reactions (ADRs) reported by the study participants (N = 42)

Figure 3 shows the distribution of ADRs reported among the 150 participants, with a total of 42 events recorded. Akathisia was the most common ADR, reported in nine (21.4%) cases, followed by tremors and drowsiness in six (14.3%) each. Other notable reactions included weakness in five (11.9%), tardive dyskinesia and weight gain in four (9.5%) each, and less frequent events such as headache, hypersalivation, dry mouth, constipation, and throat irritation. This pattern underscores the need for careful monitoring of extrapyramidal and sedative side effects during treatment.

## Discussion

Prescription patterns among individuals with psychosis vary across regions, reflecting differences in clinical practice, drug availability, and healthcare settings. Although previous studies have highlighted ADRs to antipsychotic medications, limited data exist on ADRs in the Indian population [10,11]. Therefore, this study aimed to understand prescribing trends and the influence of medication-related side effects in psychotic disorders. Rational prescription and appropriate drug utilization are essential to ensure patient safety and optimize healthcare resources [12].

In addition to psychotropic polypharmacy, co-administration of antipsychotic medications with centrally acting analgesics such as tramadol warrants clinical caution [13]. Tramadol, an opioid analgesic with serotonergic and dopaminergic properties, may interact with antipsychotics, leading to excessive sedation, confusion, or even psychotic exacerbations [14]. Polypharmacy involving these agents can amplify central nervous system depression and cognitive impairment [15]. A recent systematic review highlighted that tramadol use has been associated with psychological side effects, including hallucinations, mood changes, and psychosis [16]. Hence, concurrent use of antipsychotics and tramadol should be closely monitored to prevent neuropsychiatric complications.

In the present study, the mean age of patients was 36.4 ± 11.5 years, with most (34.7%) aged between 18 and 30 years. The gender distribution (43.3% male, 56.7% female) aligns with previous Indian findings, suggesting equal disease prevalence among the sexes [17,18]. Psychosis NOS was the most common diagnosis (42.7%), followed by schizophrenia (30%), bipolar affective disorder (21.3%), depression with psychotic symptoms (4%), and organic psychosis (2.9%). Atypical antipsychotics predominated (88.8%), consistent with current global trends favoring these drugs over typical agents due to improved safety profiles [19].

Risperidone (42%) and olanzapine (27.3%) were the most frequently prescribed antipsychotics, followed by aripiprazole and haloperidol (each 9.3%). Similar prescribing trends have been reported in Indian studies [20,21]. The mean number of drugs per encounter (2.69 ± 0.84) exceeded the WHO-recommended range (1.6-1.8), suggesting mild polypharmacy. This likely reflects the need for adjunct medications to manage comorbidities or counteract side effects.

Only 46.3% of prescriptions used generic names, lower than the WHO’s recommended 100% standard, indicating reliance on branded drugs [22-24]. Economic constraints often drive such choices, as prescribers perceive branded drugs to be more reliable [25]. Promoting generic prescribing could help reduce healthcare costs and medication errors. Injectable formulations, mainly risperidone and haloperidol, were used selectively for noncompliant patients, aligning with rational prescribing practices [26,27]. No antibiotics were prescribed, consistent with psychiatric care norms [28].

About 72.5% of drugs were from the NLEM (2022), below the WHO ideal of 100% but comparable to other studies [29]. Increased adherence to essential medicine lists can enhance rational drug use. Polypharmacy involving multiple antipsychotics was observed in 8.7% of encounters, similar to other Indian studies [30]. However, international data show higher rates, ranging from 45.7% in Southeast Asia to 61.5% in India’s Nagpur region [31]. The lower rate in this study may reflect outpatient sampling and the predominance of atypical antipsychotics.

While polypharmacy can be justified in treatment-resistant cases, it also increases the risk of interactions and ADRs. Psychotropic drugs acting on the central nervous system often cause adverse reactions, and concomitant use of anticholinergics to counteract extrapyramidal effects can introduce additional risks such as constipation, dry mouth, and photophobia [27]. The relatively lower anticholinergic use in this study likely reflects limited extrapyramidal side effects due to widespread use of atypical agents (88.8%) [29]. Nevertheless, long-term prophylactic use of anticholinergics remains a concern and should be minimized.

The overall ADR incidence in this study was 28%, involving 42 reported events among 150 patients. The most common ADRs were akathisia (21.4%), drowsiness (14.3%), tremors (14.3%), and weakness (11.9%), followed by tardive dyskinesia, weight gain, headache, and hypersalivation. Similar ADR profiles were reported in previous Indian studies [32]. Risperidone and olanzapine were most frequently associated with ADRs, consistent with their higher prescription rates. Based on the WHO-UMC causality assessment, 90.5% of ADRs were classified as “possible” and 9.5% as “probable,” aligning with comparable findings in other reports [33].

The cross-sectional nature of this study limits causal inference and the ability to assess longitudinal trends or treatment outcomes. Being a single-center study also restricts generalizability. ADR detection relied on spontaneous reporting, which may have led to underestimation of minor or delayed reactions. Furthermore, no severity grading or rechallenge testing was conducted, which could have strengthened causality assessment. Future multicentric prospective studies with larger sample sizes, continuous ADR monitoring, and pharmacoeconomic analyses are warranted to validate these findings and support rational psychotropic prescribing in India.

## Conclusions

The results of the present research indicate that the most commonly prescribed antipsychotic was risperidone, and the predominance of atypical antipsychotics reflects their favorable clinical and safety profile. Although polypharmacy appeared infrequent in this study, this finding may be influenced by the outpatient setting, as inpatients, who are more likely to receive multiple concurrent antipsychotics, were excluded from analysis. A significant proportion of prescriptions included concomitant medications such as clonazepam and trihexyphenidyl, primarily for managing anxiety and extrapyramidal symptoms, respectively.

Only 69 (46.3%) prescriptions were written using generic names, indicating suboptimal adherence to WHO recommendations. Physicians should be encouraged to prioritize generic prescribing to promote rational and cost-effective drug use. The present study provides valuable insights into prescribing trends, highlighting the incidence and spectrum of ADRs detected in outpatient psychiatric practice. Continued efforts are required to standardize treatment protocols for psychotic disorders, strengthen pharmacovigilance systems, and enhance prescriber awareness regarding rational psychotropic use. Regular patient counseling and routine monitoring of medication responses should be emphasized to ensure safe and effective long-term therapy.
